# TREX1 degrades the 3′ end of the small DNA oligonucleotide products of nucleotide excision repair in human cells

**DOI:** 10.1093/nar/gkac214

**Published:** 2022-03-31

**Authors:** Seon Hee Kim, Geun Hoe Kim, Michael G Kemp, Jun-Hyuk Choi

**Affiliations:** Biometrology Group, Division of Chemical and Biological Metrology, Korea Research Institute of Standards and Science, Daejeon 305-340, Republic of Korea; Department of Bio-Analytical Science, University of Science & Technology, Daejeon 305-340, Republic of Korea; Biometrology Group, Division of Chemical and Biological Metrology, Korea Research Institute of Standards and Science, Daejeon 305-340, Republic of Korea; Department of Bio-Analytical Science, University of Science & Technology, Daejeon 305-340, Republic of Korea; Department of Pharmacology and Toxicology, Wright State University Boonshoft School of Medicine, Dayton, OH 45435, USA; Dayton Veterans Administration Medical Center, Dayton, OH 45428, USA; Biometrology Group, Division of Chemical and Biological Metrology, Korea Research Institute of Standards and Science, Daejeon 305-340, Republic of Korea; Department of Bio-Analytical Science, University of Science & Technology, Daejeon 305-340, Republic of Korea

## Abstract

The nucleotide excision repair (NER) machinery removes UV photoproducts from DNA in the form of small, excised damage-containing DNA oligonucleotides (sedDNAs) ∼30 nt in length. How cells process and degrade these byproducts of DNA repair is not known. Using a small scale RNA interference screen in UV-irradiated human cells, we identified TREX1 as a major regulator of sedDNA abundance. Knockdown of TREX1 increased the level of sedDNAs containing the two major UV photoproducts and their association with the NER proteins TFIIH and RPA. Overexpression of wild-type but not nuclease-inactive TREX1 significantly diminished sedDNA levels, and studies with purified recombinant TREX1 showed that the enzyme efficiently degrades DNA located 3′ of the UV photoproduct in the sedDNA. Knockdown or overexpression of TREX1 did not impact the overall rate of UV photoproduct removal from genomic DNA or cell survival, which indicates that TREX1 function in sedDNA degradation does not impact NER efficiency. Taken together, these results indicate a previously unknown role for TREX1 in promoting the degradation of the sedDNA products of the repair reaction. Because TREX1 mutations and inefficient DNA degradation impact inflammatory and immune signaling pathways, the regulation of sedDNA degradation by TREX1 may contribute to photosensitive skin disorders.

## INTRODUCTION

A variety of DNA damaging agents ranging from environmental carcinogens to anti-cancer drugs induce the formation of bulky lesions on DNA that can only be targeted for removal by the nucleotide excision repair (NER) machinery in human cells (1). This multi-enzyme system involves damage recognition, unwinding of the DNA around the lesion, and nucleolytic incisions on the damaged strand of DNA by structure-specific endonucleases. The dual incision reaction therefore releases the damaged nucleotides from the genome in the form of a small, excised, damage-containing DNA oligonucleotide (sedDNA) (2–4). The ssDNA gap that remains in the duplex is filled in by DNA synthesis and ligation to complete the repair reaction (5).

Though this model of NER is reasonably well understood, the fate of the sedDNA products of NER is not clear and has not been extensively examined (6). Studies with cell-free extracts and purified proteins in vitro revealed that the sedDNAs are initially stably associated with the NER factor TFIIH before being released in an ATP-dependent but ATP hydrolysis-independent manner and become bound to the single-stranded DNA (ssDNA)-binding protein RPA (Replication Protein A) (7). Studies with UV-irradiated cultured cells have also demonstrated that sedDNAs associate with both TFIIH and RPA in cultured cells in vivo (3,4). Furthermore, inhibiting the gap filling synthesis and/or ligation steps of NER, which is known to slow UV photoproduct removal from the genome (8–11) and prevent RPA re-localization to new sites of UV damage (12), was found to result in a modest accumulation of RPA-bound sedDNAs (4). Though studies examining the kinetics of NER and sedDNA abundance have observed that sedDNA levels begin to decrease 2–4 h after UV exposure and correlate in time with the induction of apoptotic signaling at high UV doses, sedDNA levels were not affected by caspase inhibition and thus are not likely degraded by apoptotic nucleases (13). Thus, significant questions remain regarding the processing and degradation of sedDNAs and whether the association of sedDNA proteins with TFIIH and XPA impact the rate of damage removal from the genome or the whether inefficient sedDNA degradation contributes to aberrant activation of cytosolic DNA sensor proteins involved in inflammation and autoimmunity (14–16).

## MATERIALS AND METHODS

### Cell culture and transfections

HeLa cells and A375 cells were obtained from the Korean Cell Line Bank of Seoul National University (Seoul, Korea) and the American Type Culture Collection (Rockville, MD, USA). The cells were cultured in Dulbecco's modified Eagle's medium (Gibco) supplemented with 10% fetal bovine serum at 37°C in a 5% CO_2_ humidified incubator. For small RNA interference screens, the following SMARTpool ON-TARGETplus siRNA reagents were purchased from Dharmacon: Non-Targeting siControl (D-001206-13-20), siXPF (M-019946-00-0005), siEXO1 (M-013120-00-0005), siSNM1A (M-010790-00-0005), siSNM1B (M-015780-00-0005), siArtemis (M-004269-02-0005), siTrex1 (M-013239-03-0005), siTrex2 (M-032280-02-0005), siMus81 (M-016143-01-0005), siMre11 (M-009271-01-0005), siCtIP (M-011376-00-0005), siAPEX1 (M-010237-01-0005), siGEN1 (M-018757-01-0005), siFEN1 (M-010344-01-0005), siWRN (M-010378-01-0005), siNME1 (M-006821-01-0005), siDNASE1 (M-016280-01-0005), siDNASE2 (M-009667-01-0005), siEXO5 (M-014212-00-0005), siSLX4 (M-014895-01-0005), siDNA2 (M-026431-01-0005). Where indicated, a single siRNA targeting Trex1 (CCAAGACCAUCUGCUGUCA) was used in comparison with Non-Targeting siRNA (UAGCGACUAAACACAUCAA), as previously used (Yan *et al.*, Nat. Immunol. 2010). Transfections were performed using DharmaFECT I transfection reagent (Dharmacon) or Lipofectamine RNAiMAX transfection reagent (ThermoFisher), according to the manufacturer's instructions. The mammalian expression plasmids expressing GFP-Trex1 (Plasmid #27219), GFP-Trex1(D18N) (Plasmid #27220) and Flag-Trex1 (Plasmid #27218) that had been previously used (Yan *et al.*, Nat. Immunol. 2010) were obtained from Addgene. Transfections were performed using Lipofectamine 2000 transfection reagent (Thermo Fisher Scientific), according to the manufacturer's instructions. Cells were exposed to a 254 nm UVC light source and harvested at the indicated time points.

### sedDNA detection and quantification

Cells were lysed in cold Triton X-100 lysis buffer (20 mM Tris–HCl, pH7.5, 150 mM NaCl, 1 mM EDTA, 1 mM EGTA and 1% Triton X-100) and incubated on ice for 20 min with occasional vortexing. Following centrifugation for 1 h at maximum speed in a microcentrifuge at 4°C to pellet genomic DNA, the soluble lysates were digested with RNase A (10–20 μg/ml) and RNase T1 (30–50 units/ml) for 20 min at 37°C, and then treated with 0.15 mg/ml of proteinase K in the presence of 0.4% SDS for 30 min at 55°C. The reactions were subsequently extracted with phenol/chloroform and precipitated in ethanol. After centrifugation, the pellets were washed with 70% ethanol, resuspened in Tris-EDTA buffer (10 mM Tris–HCl pH 8.0, 1 mM EDTA). Where indicated, the samples were subjected to immunoprecipitation with anti-(6-4)PP or anti-CPD antibody as described previously (17–19). Briefly, the samples were immunoprecipitated with Dynabeads bound to anti-(6-4)PP or anti-CPD antibody (Cosmo Bio), washed with Wash Buffer I (20 mM Tris–HCl pH 8.0, 2 mM EDTA, 150 mM NaCl, 1% Triton X-100 and 0.1% SDS), Wash Buffer II (20 mM Tris–HCl pH 8.0, 2 mM EDTA, 500 mM NaCl, 1% Triton X-100, 0.1% SDS), Wash Buffer III (10 mM Tris–HCl pH 8.0, 1 mM EDTA, 150 mM LiCl, 1% Nonidet P-40, 1% sodium deoxycholate), Wash Buffer IV (100 mM Tris–HCl pH 8.0, 1 mM EDTA, 500 mM LiCl, 1% Nonidet-P40, 1% sodium deoxycholate), and twice with Tris-EDTA buffer. The photoproduct-containing sedDNAs were eluted twice with elution buffer (50 mM NaHCO_3_, 1% SDS, and 20 μg.ml glycogen) at 65°C for 15 min, extracted with phenol-chloroform, precipitated in ethanol, and dried. Where indicated, cells were lysed in cold NP-40 lysis buffer (25 mM HEPES, pH 7.9, 100 mM KCl, 12.5% Glycerol, 0.5% NP-40, 0.5 mM EDTA), subjected to immunoprecipitation of RPA or TFIIH essentially as described previously (Kemp *et al.*, JBC 2012; JBC 2014). Briefly, cell extracts were incubated with anti-XPB (Santa Cruz Biotechnologies) or anti-RPA70 (Bethyl Laboratories) antibody for 4 h and were then incubated with protein A/G magnetic beads (Thermo Fisher Scientific) for 4 h. Immunoprecipitates were washed with NP-40 lysis buffer and incubated for 30 min at 55°C in elution buffer (45 mM Tris–HCl pH 8.0, 250 mM NaCl, 9 mM EDTA, 0.5% SDS and 0.4 μg.ml proteinase K). The eluted DNAs were extracted with phenol-chloroform, precipitated in ethanol, and dried.

The purified DNAs were then subjected to 3′-end labeling, gel electrophoresis, transfer to membrane, and chemiluminescence detection as described previously (17,18). Briefly, DNAs were labeled with terminal deoxynucleotidyl transferase (New England Biolabs) and biotin-11-dUTP. Reactions were stopped with 10 mM EDTA, and DNAs were treated with RNase A (10–20 μg/ml) and RNase T1 (30–50 units/ml) for 20 min at 37°C and incubated with proteinase K (0.4 mg/ml) in the presence of 0.4% SDS. Following extraction with phenol-chloroform and precipitation in ethanol, biotin-labeled DNAs were separated on 10–12% TBE-urea gels, transferred to a modified nylon Biodyne B membrane (Thermo Fisher Scientific), and then fixed by UV crosslinking. The membrane was incubated with PBS containing 2% SDS for 30 min and then incubated for 30 min with HRP-conjugated streptavidin in the same buffer. Membranes were next washed three times with PBS containing 0.5% SDS and then incubated with 200 mM Tris–HCl (pH 9.0) containing 10 mM MgCl_2_ for 5 min. Chemiluminescence was visualized with ECL reagents (GE Healthcare) on an ImageQuant LAS 4000 Mini apparatus (GE Healthcare) and quantified with ImageQuantTL software (GE Healthcare).

### Immunoslot blot assay

Following UV irradiation, cells were harvested at the time points indicated, and genomic DNA was isolated and analyzed by immunoslot blotting with anti-(6-4)PP and CPD antibodies as described previously (18,20).

### Protein immunoblotting

Cells were lysed in cold Triton X-100 lysis buffer (20 mM Tris–HCl, pH7.5, 150 mM NaCl, 1 mM EDTA, 1 mM EGTA and 1% Triton X-100) or RIPA buffer (25 mM Tris–HCl, pH 7.6, 150 mM NaCl, 1% NP-40, 1% sodium deoxycholate and 0.1% SDS) and analyzed by immunoblotting as previously described (13,18) using antibodies against TREX1 (Cell Signaling Technology), GAPDH (Cell Signaling Technology), GFP (Thermo Fisher Scientific), Flag epitope (Sigma).

### *In vitro* nuclease assay

For purification of GFP-fusion proteins, A375 cells grown in 60 mm diameter plates were transfected with vectors expressing GFP alone, GFP-tagged TREX1 or GFP-tagged D18N using Lipofectamine 2000 transfection reagent. The cells were harvested 48–72 h later and lysed in cold NP-40 lysis buffer (25 mM HEPES, pH 7.9, 100 mM KCl, 12.5% Glycerol, 0.5% NP-40, 0.5 mM EDTA) containing 1X protease inhibitor cocktail (Roche Applied Science) for 30 min at 4°C on a rotating shaker. Following centrifugation for 1 h at maximum speed in a microcentrifuge at 4°C, the soluble lysates were incubated with 1 μg of anti-GFP antibody (Thermo Fisher Scientific) for 16 h at 4°C on a rotating shaker and further incubated with 20 μl of protein A/G magnetic beads (Thermo Fisher Scientific) for 4 h. The beads were washed with cold NP-40 lysis buffer three times, and the immunoprecipitated proteins were eluted from the beads in IP elution buffer (0.1 M Glycine-HCl, pH 2.5). The eluted proteins were immediately neutralized by adding 1/10 volume of 1 M Tris–HCl (pH 9.0).

For isolation of sedDNA substrates, A375 cells grown to near confluency in 150 mm diameter plates were exposed to 20 J/m^2^ of UVC radiation and harvested 1 h later. The cells were lysed in a 10× packed cell volume of cold Triton X-100 lysis buffer and incubated for 30 min at 4°C on a rotating mixer. Soluble cell lysates were prepared by centrifugation at 40 000 *× g* in a superspeed centrifuge (Sorvall RC 6 Plus, Thermo Fisher Scientific) for 1 h at 4°C and collected in new tubes. The cell lysates were incubated with RNase A (10 μg/ml) and RNase T1 (20 units/ml) for 20 min at 37°C, treated with 0.15 mg/ml of proteinase K for 30 min at 55°C. The reactions were extracted with phenol/chloroform and precipitated in ethanol. Following centrifugation, the pellets were washed with 70% ethanol twice and resuspended in Tris-EDTA buffer (10 mM Tris–Cl, pH 8.0, 1 mM EDTA). To remove any trace of contaminating RNA, the DNA preparations were incubated with RNase A (2 μg/ml) and RNase T1 (4 units/ml) in the presence of 100 mM NaCl for 30 min at 37°C and then extracted with phenol/chloroform. Following ethanol precipitation, the pellets were resuspended in Tris–EDTA buffer and subjected to nuclease assays.

In some experiments, a defined 30-mer oligonucleotide (5′- GAAGGAAGGAAGGAAGGAAGGAATTAAGGA) lacking or containing a 5′-biotin was exposed to 254 nm UV radiation to generate model DNA substrates containing a single, defined UV photoproduct. DNAs were subjected to immunoprecipitation with anti-CPD or anti-(6-4)PP antibodies as described above to isolate specific photoproduct-containing DNAs prior to digestion with TREX1. Nuclease assay reaction mixtures contained 15 mM Hepes pH 7.9, 60 mM KCl, 7.2 mM MgCl_2_, 7.5% Glycerol, 0.3% NP-40 and purified sedDNAs (0.5 fmol/μl) and GFP fusion proteins (0.5 ng/μl) unless otherwise indicated in the figure. The mixtures were incubated for 1 h at 37°C, unless otherwise indicated, and the reactions were terminated by the addition of 10 mM EDTA on ice. The reactions were incubated with 10 μg of proteinase K in the presence of 0.4% SDS at 30 min for 55°C, extracted with phenol/chloroform, and precipitated in ethanol. The extracted DNA samples were then subjected to labeling, gel electrophoresis, membrane transfer, and chemiluminescence detection as described above.

### Statistical analyses

Statistical analysis was performed using GraphPad Prism 7 software, and two-tailed, paired or unpaired Student's t-tests were carried out where indicated to determine whether differences between treatment samples reached statistical significance (*P* < 0.05).

## RESULTS

### RNAi screen for novel regulators of sedDNA abundance

**Figure 1.**
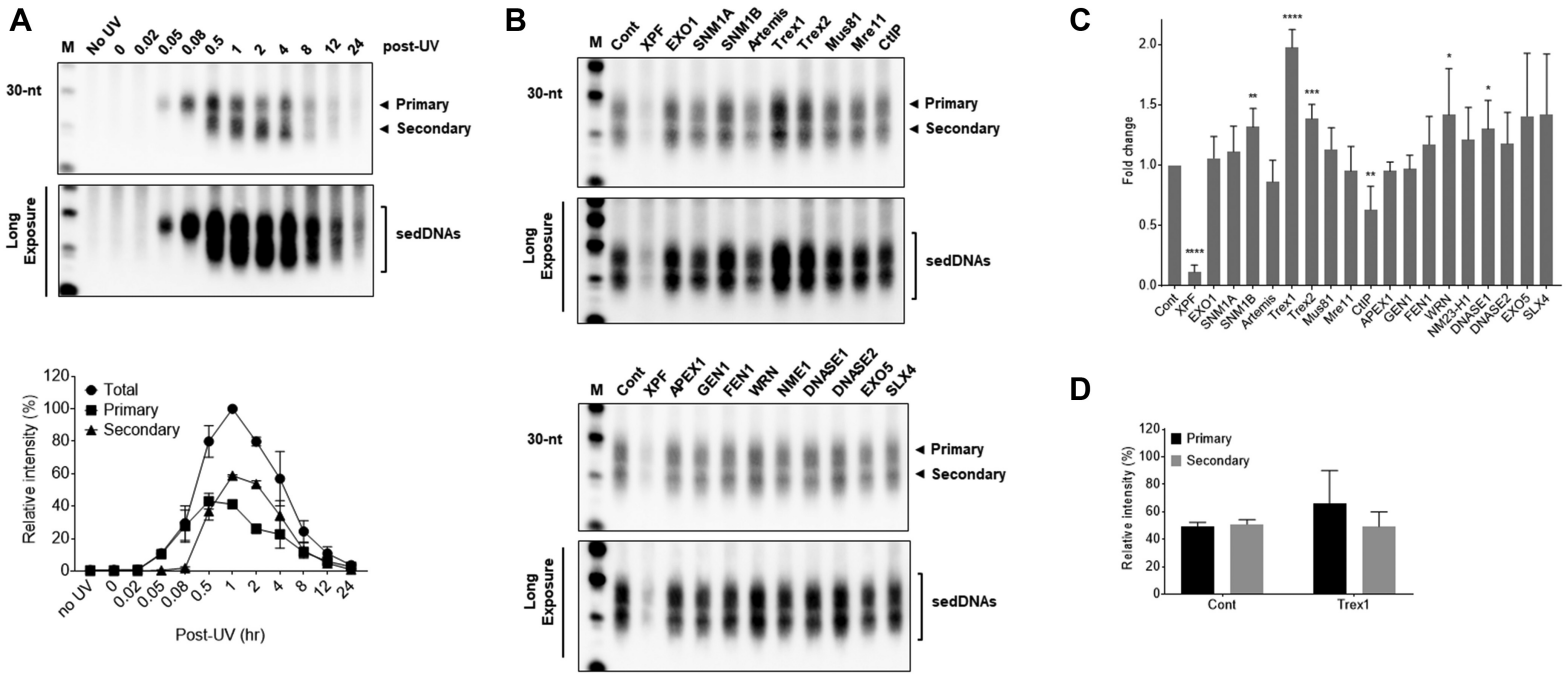
RNA interference screen to identify novel regulators of sedDNA abundance. (**A**) HeLa cells were exposed to 20 J/m^2^ UV, harvested at the indicated time points, and then sedDNA products of NER were visualized as described in the Materials and Methods section. The primary and secondary, partially degraded populations of sedDNAs are indicated. (**B**) Representative results from RNAi screen in which sedDNAs were isolated and detected in HeLa cells transfected with the indicated siRNA pool and exposed to 10 J/m^2^ UV. (**C**) Quantitation of results (average and SEM) from five independent experiments performed as in (B). A one-sided t-test was used to determine whether knockdown of specific nucleases impacted sedDNA levels in comparison to the control transfection (*P < 0.05; **P < 0.01; ***P < 0.001; ****P < 0.0001). (**D**) Quantitation of the percentage of primary and secondary sedDNAs in cells transfected with control and Trex1 siRNA. (D) Quantitation of the percentage of primary and secondary sedDNAs in cells transfected with control and Trex1 siRNA.

Human cells respond rapidly to UV exposure and DNA damage by removing UV photoproducts from the genome in the form of sedDNAs (17). As shown in Figure 1A, sedDNAs can be observed in UV-irradiated HeLa cells within 3 min of exposure. At early time points, the sedDNAs are primarily in the form of a larger species of DNAs nearly 30 nt in length (referred to as primary products). By 30 min after exposure, a second, partially degraded population approximately 20 nt in length can also be observed. Studies with the minimal set of six purified NER factors sufficient for dual incisions in vitro have demonstrated that only the primary products are observed (7), which indicates that conversion to the secondary, partially degraded form is likely due to the action of one or more other cellular nucleases. Consistent with previous reports (17,18), total sedDNA abundance peaks at 1 hr after UV exposure and then begins to decrease at later time points.

To begin to explore how the sedDNA products of NER are degraded in human cells, a small scale genetic screen was performed in which HeLa cells were transfected with siRNA pools targeting 20 known DNases and then exposed to 10 J/m^2^ of UV radiation. Cells were incubated for 1 h and then harvested for sedDNA detection. Representative results of this screening are shown in Figure 1B, and quantification of sedDNA levels from 5 independent experiments relative to cells transfected with a control siRNA pool are shown in Figure 1C. As expected, knockdown of XPF, which generates the 5′ incision during NER, reduced sedDNA levels by up to 10-fold. Interestingly, knockdown of CtIP, which is known to function in the resection of DNA double-strand breaks (21,22), led to a small, ∼35% reduction in sedDNA levels. Knockdown of most of the other screened nucleases had either no effect on sedDNA abundance or was associated with small increases of less than 50%. In contrast, knockdown of Trex1 led to a reproducible 2-fold increase in sedDNA levels (Figure 1C) but did not impact the size distribution (Figure 1D). Trex1 is the major 3′-exonuclease in human cells and is mutated in certain patients with autoimmune disorders (23–25).

### Knockdown of Trex1 increases sedDNA abundance in human cells exposed to UV radiation and other chemical carcinogens

**Figure 2.**
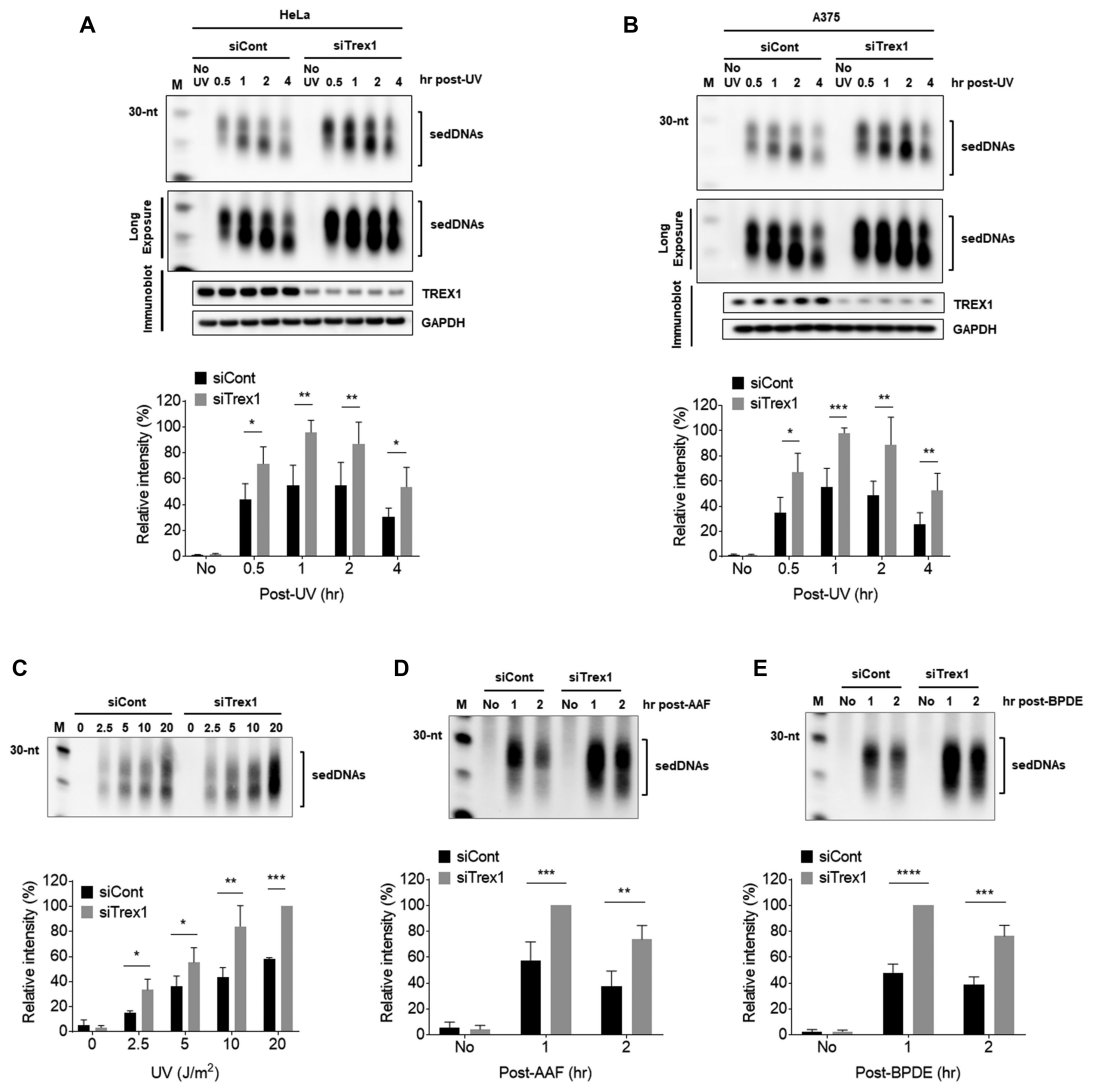
Knockdown of Trex1 in human cells increases sedDNA levels in cells exposed to UV radiation and chemical carcinogens. (**A**) HeLa cells were transfected with in the indicated siRNA, exposed to 20 J/m^2^ UV, and then harvested at the indicated time points for analysis of sedDNAs. (**B**) A375 melanoma cells were treated as in (A). (**C**) A375 cells were transfected as in (A) and exposed to the indicated fluences of UV radiation. Cells were harvested 1 h later for analysis of sedDNAs. (D, E) HeLa cells transfected as in (A) were exposed to either *N*-acetyoxy-2-acetylaminofluorene (AAF) or benzo[a]pyrene diol epoxide (BPDE) and then harvested at the indicated time points to detect the sedDNA products of NER. All graphs show the relative level of sedDNAs from at least three independent experiments. *T*-tests were used to compare the relative sedDNA abundance at each time point and UV dose.

To confirm the results of this screen, HeLa cells were transfected with a single siRNA targeting Trex1, exposed to UV radiation, and then harvested at various time points for analysis of sedDNA levels. As shown in Figure 2A, sedDNA levels were increased by up to two-fold after UV exposure. Similar results were observed in A375 melanoma cells (Figure 2B). The effect of Trex1 knockdown on increasing sedDNA levels was also found to occur in A375 cells exposed to a range of UV fluences (Figure 2C). Finally, the abundance of sedDNAs generated following UV exposure were ∼2.5-fold higher in mouse embryonic fibroblasts from Trex1-knockout mice in comparison to wild-type cells (Supplementary Figure S1).

Knockdown of the related nuclease 3′-exonuclease Trex2 in HeLa cells was also associated with a modest, nearly 40% increase in sedDNA abundance (Figure 1C). However, knockdown of Trex2 in UV-irradiated A375 melanoma cells had no effect on sedDNA levels (Supplementary Figure S2), and co-depletion of both Trex1 and Trex2 did not increase sedDNA levels more than upon knockdown of Trex1 alone in these cells. Thus, Trex1 appears to be the major nuclease that impacts the stability of sedDNAs in UV-irradiated cells.

Like UV radiation, the chemical carcinogens *N-*acetyoxy-2-aminofluorene (AAF) and benzo[a)pyrene diol epoxide (BPDE) generate bulky DNA lesions that are targeted for removal by the NER machinery. Treatment of HeLa cells depleted of Trex1 with either AAF or BPDE led to similar, nearly two-fold increases in sedDNA abundance in comparison to cells transfected with control siRNA (Figure 2D, E). Thus, the loss of Trex1 is associated with increased sedDNA levels in response to diverse agents that induce DNA adducts repaired by NER.

**Figure 3.**
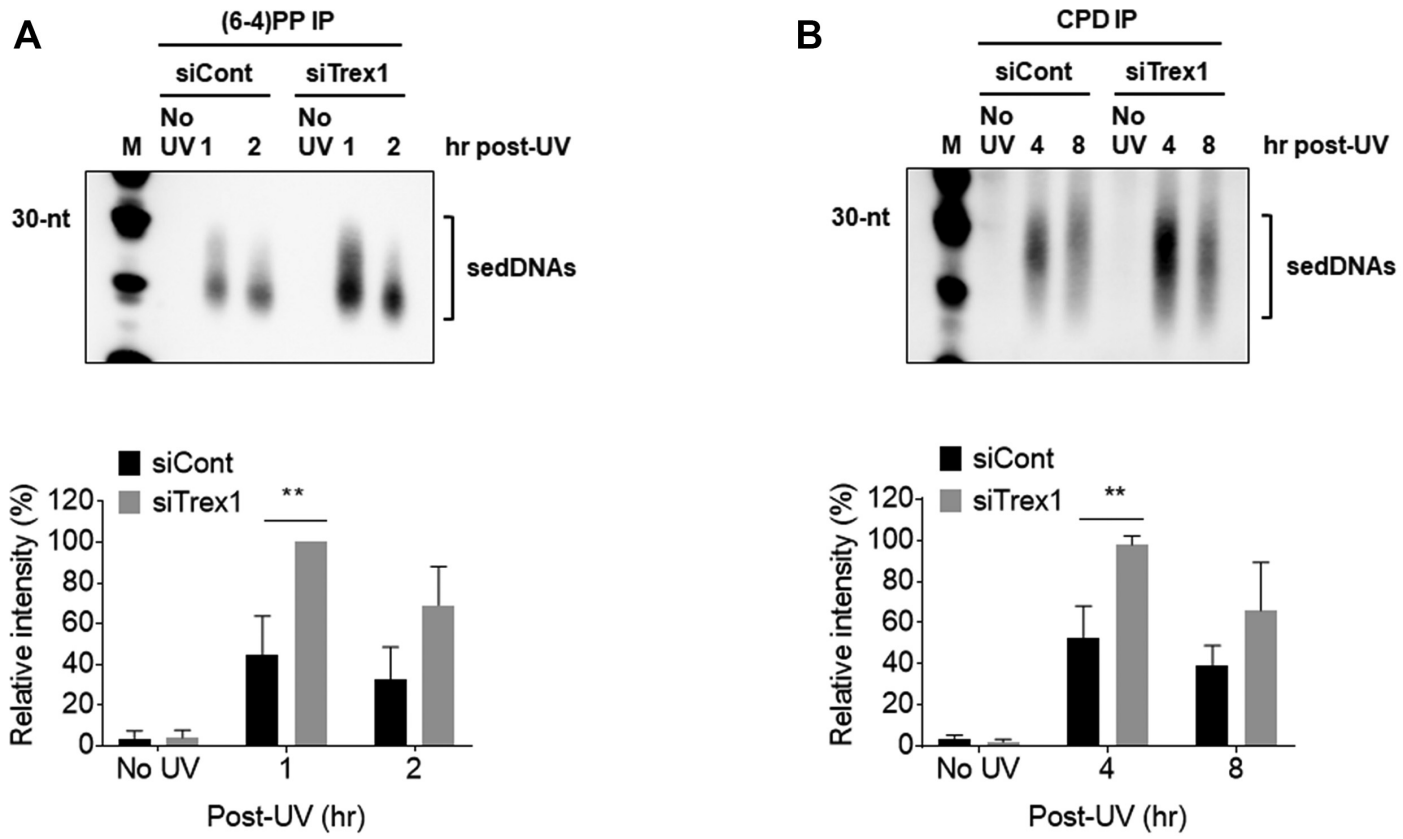
Knockdown of Trex1 increases the levels of (6-4)PP- and CPD-containing sedDNAs. (**A**) HeLa cells were treated as in Figure 2 except that sedDNAs were immunoprecipitated with an antibody against (6-4)PPs. (**B**) Cells were processed as in (A) except that an anti-CPD antibody was used for immunoprecipitation. The graphs show the relative level of sedDNAs from at least three independent experiments.

The two major lesions induced in DNA by UV radiation are pyrimidine (6-4) pyrimidone photoproducts [(6-4)PPs] and cyclobutane pyrimidine dimers (CPDs) (26). To explore whether sedDNAs containing either of these lesions are differentially affected by knockdown of Trex1, antibodies that specifically recognize (6-4)PPs or CPDs were used to isolate the respective populations of sedDNAs after purification from UV-irradiate cells. For each class of photoproduct-containing sedDNAs, cells were exposed to UV radiation and then harvested at time points that correspond to maximum and reduced abundance (13,17). As shown in Figure 3A and B, both (6-4)PP- and CPD-containing sedDNA levels were increased in cells transfected with the Trex1 siRNA. However, a slightly larger difference was observed for the (6-4)PP-containing sedDNAs. Whether this modest difference is due to their differential rate of repair by the NER machinery, inherent properties of the different photoproduct-containing sedDNAs, or the time after UV exposure is not clear.

**Figure 4.**
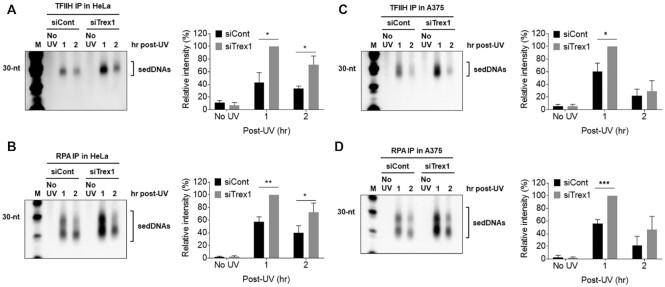
Knockdown of Trex1 increases sedDNA association with the NER factors TFIIH and RPA. (**A**) HeLa cells were treated as in Figure 2 except that cell lysates were immunoprecipitated with an anti-TFIIH antibody. (**B**) Cell lysates from cells treated as in (A) were immunoprecipitated with an anti-RNA antibody. (**C**, **D**) A375 cells were processes as for HeLa cells in (A) and (B). Graphs show the average level of TFIIH- and RPA-bound sedDNA from three independent experiments.

The sedDNA products of NER are also known to associate with two protein complexes (TFIIH and RPA) following the dual incision reaction (3,7). To determine whether Trex1 specifically impacts the association of sedDNAs with either of these proteins, HeLa cells were transfected with control or Trex1 siRNAs, exposed to UV, and harvested after 1 or 2 h for the preparation of cell lysates. Antibodies targeting TFIIH and RPA were then used to isolate the respective protein-sedDNA complex. As shown in Figure 4A, approximately 2- to 2.5-fold more sedDNAs were found to be in complex with TFIIH in cells in which Trex1 was knocked down. A similar effect was observed for RPA-bound sedDNAs (Figure 4B) and in A375 cells (Figure 4C, D). Thus, loss of Trex1 is associated with increased sedDNA association with both TFIIH and RPA.

### Trex1 overexpression decreases sedDNA abundance in UV-irradiated human cells

**Figure 5.**
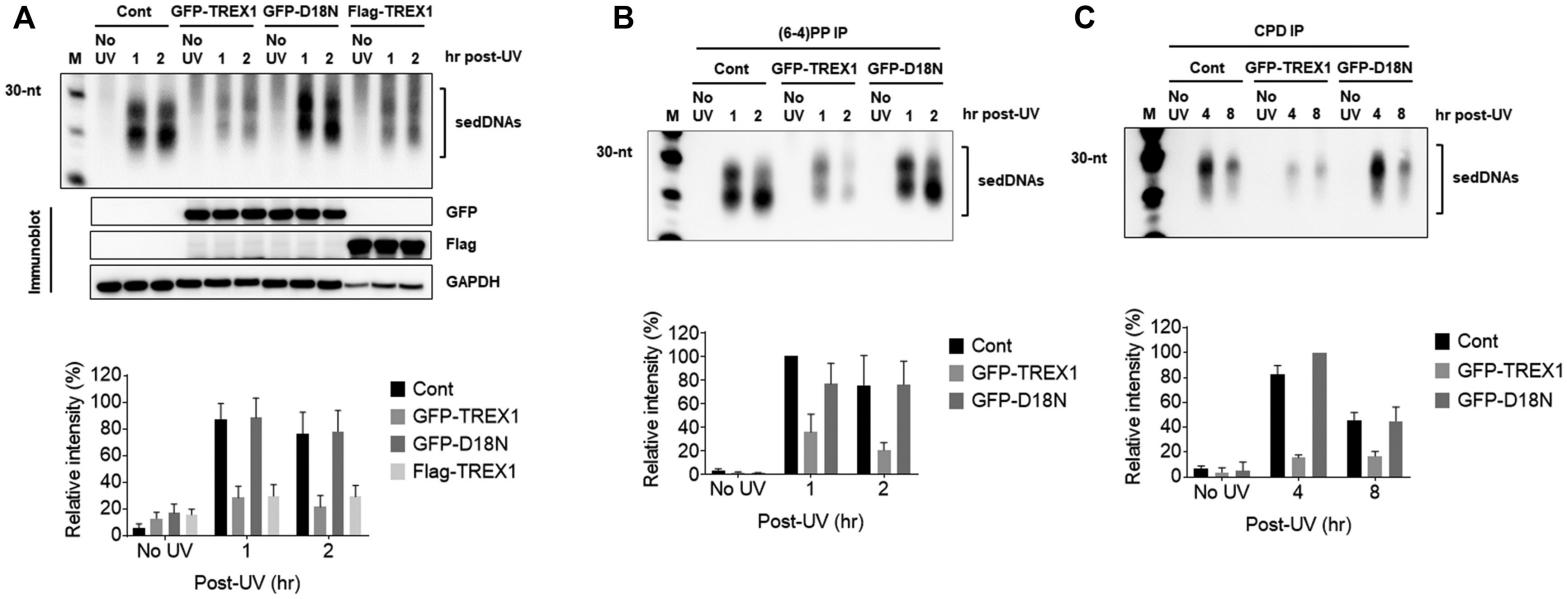
Overexpression of Trex1 decreases sedDNA abundance in UV-irradiated cells. (**A**) HeLa cells were transfected with expression vectors expressing the indicated fusion proteins, exposed to UV, and then harvested for analysis of sedDNAs. Quantitation of relative sedDNAs from five independent experiments. (**B**, **C**) Cells were processed as in (A) except that sedDNAs were purified and then immunoprecipitated with anti-(6-4)PP or anti-CPD antibodies.

To further examine how modulating Trex1 levels impacts sedDNA abundance, HeLa cells were transfected with vectors expressing either GFP- or FLAG-tagged Trex1 and then exposed to UV. As shown in Figure 5A, overexpression of both forms of Trex1 was associated with dramatically reduced sedDNA levels. Importantly, overexpression of a dominant negative form of Trex1 (D18N) failed to impact sedDNA levels (Figure 5A). Similar results were observed when the specific (6-4)PP- and CPD-containing sedDNAs were examined (Figure 5B, C). Thus, these knockdown and overexpression studies complement one another to confirm a role for Trex1 in the regulation of sedDNA abundance in UV-irradiated cells.

Because our initial siRNA screen suggested that Trex2 may contribute to sedDNA stability in UV-irradiated HeLa cells (Figure 1C) but not A375 cells (Supplementary Figure S2, we also examined the effect of Trex2 overexpression on sedDNA levels. Though we could detect Trex2 protein in A375 and HeLa cells, its level appeared to be rather low (Supplementary Figure S3A). Interestingly, overexpression of Trex2 had a similar effect as overexpression of Trex1 at reducing sedDNA levels following UV exposure (Supplementary Figure S3B, C), which indicates that the absolute level of Trex1 and Trex2 protein in cells may impact the stability and detection of sedDNA in cells exposed to UV radiation.

### Recombinant Trex1 degrades the DNA located 3′ to the UV photoproduct in sedDNAs

**Figure 6.**
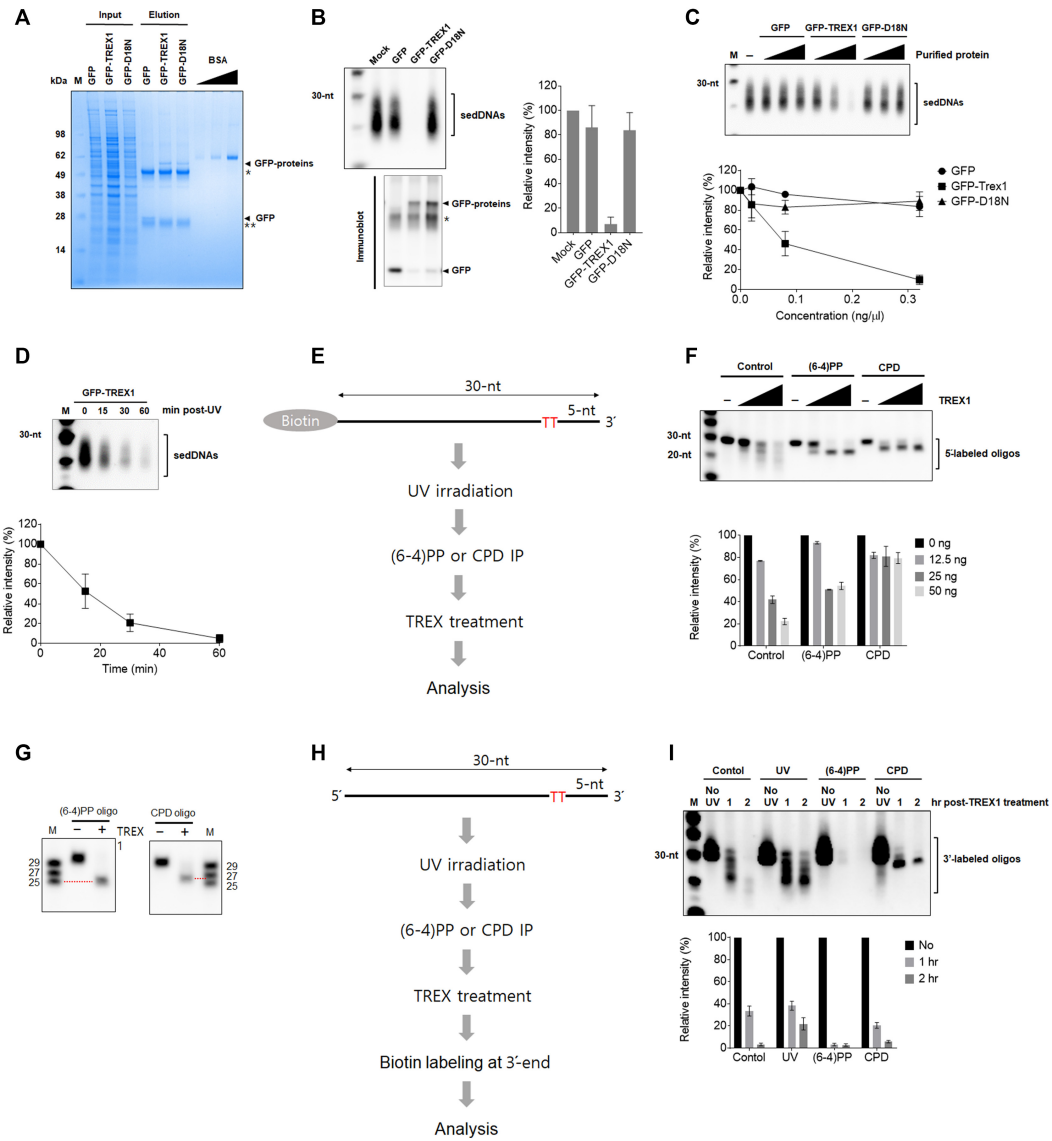
Recombinant TREX1 protein degrades the 3′ end of UV photoproduct-containing sedDNAs *in vitro*. (**A**) GFP-tagged wild-type and D18N mutant TREX1 proteins were purified from HeLa cells, separated by SDS-PAGE, and stained with Coomassie blue. The locations of the indicated proteins and antibody heavy (*) and light chains (**) are indicated. (**B**) *In vitro* nuclease assays in which the indicated recombinant proteins were mixed with sedDNAs purified from UV-irradiated cells and incubated for 1 h as described in the Materials and Methods section. Fractions of the reactions were analyzed for remaining sedDNAs (top) and for protein content by immunoblotting (bottom). (**C**) *In vitro* nuclease assays were performed as in (B) except that the reactions contained different amounts of each protein. (**D**) The reactions containing GFP-tagged wild-type TREX1 (0.16 ng/μl) were incubated with sedDNAs for the indicated time periods. All graphs show the average level of sedDNAs from at least three independent experiments. (**E**) Schematic of 30-nt-long model DNA substrate containing a single dipyrimidine sequence and a 5′ biotin. The DNA was exposed to UV radiation, subjected to immunoprecipitation with the indicated antibody, and then treated with TREX1 protein. (**F**) Analysis of model DNA substrates from (E) after digestion with recombinant TREX1. (**G**) Size analysis of (6-4)PP- and CPD-containing DNAs in (F) after digestion with TREX1. (**H**) Schematic of a second model DNA substrate lacking a 5′ biotin but treated as in (E, F) except that the DNA was 3′ end labeled with terminal transferase after digestion with TREX1. (**I**) Results from the treatment of the DNA substrate in (H) with TREX1. Note that control (non-irradiated) and UV-irradiated DNAs not subjected to immunoprecipitation with photoproduct antibodies were similarly digested with TREX1 and examined by urea PAGE and detection with HRP-streptavidin.

Recombinant wild-type and inactive (D18N) TREX1 proteins were next purified from HeLa cells for in vitro nuclease assays (Figure 6A) along with sedDNAs that were purified from UV-irradiated cells. As shown in Figure 6B, sedDNAs appeared to be completely degraded in reactions containing wild-type GFP-TREX1 but were not significantly affected by incubation with either purified GFP or mutant GFP-TREX1-D18N. Experiments with different concentrations of purified fusion proteins showed that increasing amounts of wild-type GFP-TREX1 but not GFP or GFP- TREX1-D18N resulted in a clear reduction in total sedDNA levels (Figure 6C). Kinetic experiments showed recombinant TREX1 efficiently degraded sedDNAs within a short period of time (Figure 6D).

TREX1 is a 3′ to 5′ exonuclease, and previous studies showed that TREX1 was not active on DNA substrates containing 3′ phosphates, phosphoglycolates, and tyrosyl residues (27,28). Oxidative damage to DNA has similarly been reported to inhibit degradation by TREX1 (29). Given that UV photoproducts are located 4–7 nt from the 3′ end of the sedDNA (2,3), we next examined whether UV photoproducts similarly inhibit TREX1 nucleolytic activity. We therefore exposed a 5′-biotinylated 30-mer DNA oligonucleotide with a single dipyrimidine sequence to UV radiation to generate a DNA substrate containing CPDs and (6-4)PPs located 5 nt from the 3′ end (Figure 6E), which mimics the sedDNAs generated by NER. The DNAs were then subjected to immunoprecipitation with anti-CPD or anti-(6-4)PP antibody, digested with increasing amounts of recombinant Trex1 protein, separated by urea-PAGE, transferred to nylon, and detected with streptavidin-HRP. As shown in Figure 6F, whereas the non-irradiated oligonucleotide was readily degraded to smaller species by TREX1, the digestion of the UV-irradiated substrates resulted in products of a single, defined length. Interestingly, the lengths of the digestion products of the (6-4)PP- and CPD-containing substrates were approximately 25 and 26 nt, respectively (Figure 6G). These results indicate that UV photoproducts inhibit the ability of TREX1 to completely degrade the sedDNA products of NER and that the extent of degradation is affected by the nature of the UV photoproduct, such that TREX1 becomes stalled immediately prior to (6-4)PPs but one nucleotide away from a CPD.

If UV photoproducts are located at or near the 3′ end of TREX1-degraded sedDNAs, it is possible that the photoproduct might interfere with the ability to detect such sedDNAs using our standard 3′ end labeling with terminal transferase (3,13,17–19,30). We therefore generated additional (6-4)PP- and CPD-containing DNA substrates using a similar methodology as above that lacked a 5′-biotin (Figure 6H). These substrates were then digested with TREX1 for 1–2 h, purified, and then incubated with terminal transferase and the nucleotide biotin-11-dUTP. As shown in Figure 6I, the (6-4)PP-containing reaction product was nearly undetectable whereas the CPD-containing reaction product was partially detectable but much less so than with a non-irradiated control DNA oligonucleotide that was not digested with TREX1. Interestingly, similar results were obtained when we digested a UV-irradiated, 5′-fluorescently labeled DNA oligonucleotide containing a single dipyrimidine with recombinant T4 DNA polymerase (Supplementary Figure S4), which like TREX1 has 3′ exonuclease activity that is inhibited by the presence of UV photoproducts (31). Together, these results therefore demonstrate that the presence of UV photoproducts at the 3′-terminus of ssDNAs greatly inhibits their labeling by TdT and hence their ability to be visualized. As discussed in greater detail below, these results indicate that only sedDNAs that have not been acted upon by TREX1 are detectable from UV-irradiated cells using this standard labeling methodology.

### Knockdown and overexpression of TREX1 do not impact UV photoproduct removal from genomic DNA

The apparently altered abundance of sedDNAs with Trex1 knockdown and overexpression could imply that the activity of the NER machinery in removing UV photoproducts from genomic DNA is affected by TREX1 abundance. However, a previous study showed that fibroblasts derived from individuals with Trex1 mutations were proficient in CPD removal following exposure to a solar simulating light source (32). To determine whether siRNA-mediated knockdown of Trex1 impacts NER activity throughout the genome, HeLa cells were transfected with control or Trex1 siRNA, exposed to UV radiation, and then harvested at various time points for immunoslot blot analysis of genomic DNA. As shown in Supplementary Figure S5A and B, knockdown of Trex1 did not impact the rate of removal of (6-4)PPs or CPDs from genomic DNA. To confirm these results with a different experimental methodology, immunofluorescence microscopic examination of (6-4)PPs and CPDs was performed in A375 cells and similarly did not show an effect on photoproduct removal (Supplementary Figure S6A, B). Furthermore, though a recent report suggested that Trex1 deficiency was associated with a slightly higher level of CPD induction following UV exposure (33), quantitation of the immunoslot blot data in Supplementary Figure S5 from cells harvested immediately after UV irradiation did not demonstrate any difference in either (6-4)PP or CPD formation upon Trex1 knockdown or overexpression (Supplementary Figure S6C, D).

To determine whether Trex1 overexpression impacts UV photoproduct repair, cells were next transfected with vectors expressing either GFP, wild-type GFP-Trex1, or the D18N mutant of Trex1 and then exposed to UV. However, no differences in the rate of (6-4)PP or CPD removal were observed in these cells (Supplementary Figure S5C, D). Together, these results demonstrate that though Trex1 knockdown and overexpression leads to corresponding increases and decreases in sedDNA levels following UV exposure, respectively, altered Trex1 levels do not impact the rate of removal of UV photoproducts from the genome. Moreover, knockdown of Trex1 had no effect on acute survival in UV-irradiated cells (Supplementary Figure S7).

## DISCUSSION

The removal of UV photoproducts from the genome by the NER system is critical to prevent mutagenesis and cell death. Though much is known about the general mechanism of NER (1), the fate of the UV photoproduct-containing sedDNA products of NER has not garnered as much attention (6). In *E. coli*, genetic loss of the UvrD helicase leads to a dramatic stabilization of the excised products of repair (34) and prevents their degradation by various cellular nucleases, including ExoI, ExoVII, and RecJ, and other unknown ssDNA nucleases. However, little work has been done to examine how the sedDNA products of NER are processed and degraded in mammalian cells. Given the increasingly recognized role for aberrant DNA degradation and localization in the stimulation of inflammatory and innate immune signaling pathways (15,23,25), understanding the sedDNA degradation pathway may have important implications for disease states associated with inflammation and autoimmunity. Thus, the small scale RNAi screen performed here was the first to begin to address this understudied aspect of NER.

**Figure 7.**
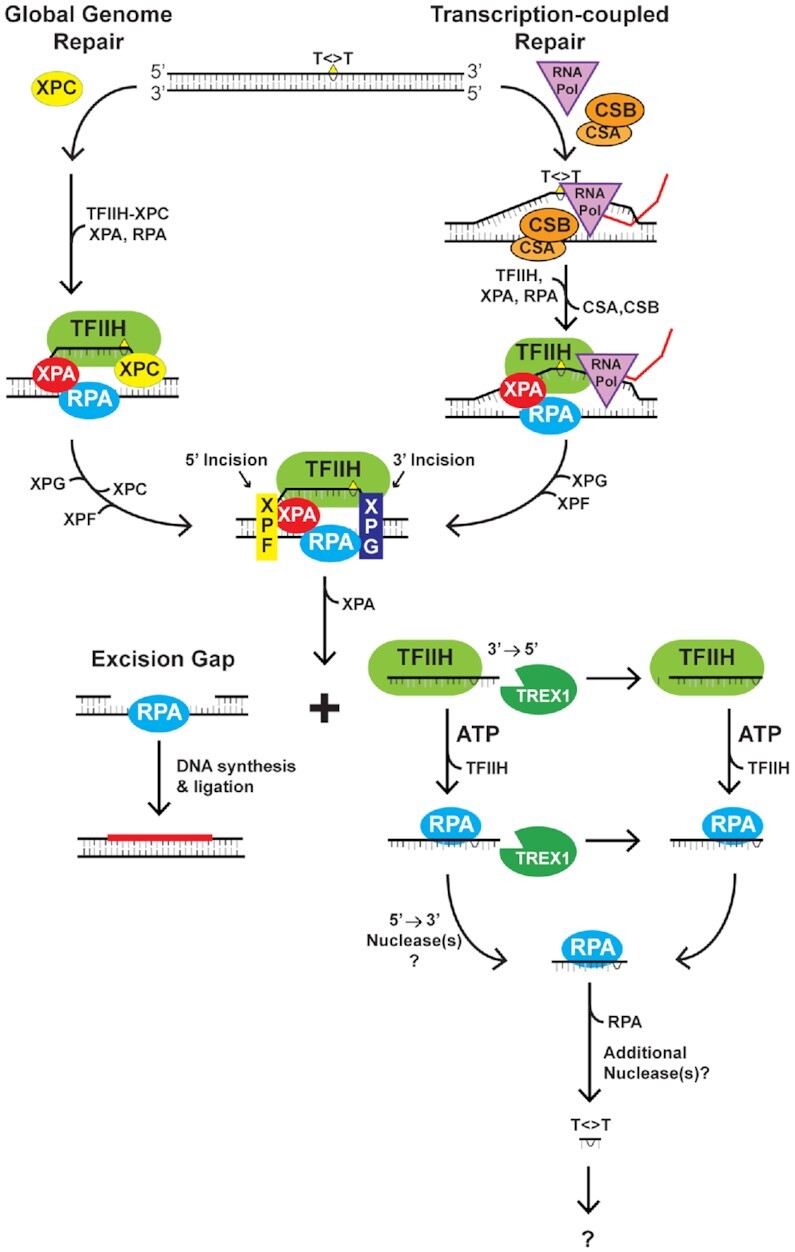
Schematic summarizing NER and the role of TREX1 in degrading the sedDNA products of the repair reaction. In response to exposure to UV radiation or other chemical carcinogens, the nucleotide excision repair machinery targets the damaged bases (indicated by T<>T) for removal via either XPC-dependent or RNA polymerase/CSA/CSB-dependent pathways. TFIIH unwinds the DNA around the lesion, which allows the XPF and XPG structure-specific nucleases to incise the damage strand 5′ and 3′ of the lesion, respectively. This dual incision releases the sedDNA from duplex DNA in complex with TFIIH. The gap can be filled in by DNA synthesis and ligation. The sedDNAs can be released from TFIIH and bind to RPA, and both the TFIIH- and RPA-sedDNA complexes are susceptible to digestion by TREX1, which degrades the nucleotides located 3′ the lesion. Because RPA-bound sedDNAs are known to be smaller on average than TFIIH-bound sedDNAs, we presume that additional 5′→3′ nucleases may partially degrade the sedDNA. Ultimately, the sedDNA likely undergoes additional degradation to smaller species. However, nothing is known about these latter steps of sedDNA processing.

A summary of our findings on the role of TREX1 in degrading the sedDNA products of NER is provided in Figure 7. As shown in Figure 1, the strongest and most consistent gene identified in the screen was the 3′ exonuclease Trex1 (24). However, knockdown of other nucleases also impacted sedDNA abundance to different extents and may exhibit cell-type specificity. For example, whereas knockdown of Trex2 in HeLa cells was associated with a modest 40% increase in sedDNAs (Figure 1), no effect was observed in A375 melanoma cells (Supplementary Figure S2). However, overexpression of Trex2 in A375 cells did lead to reduced stabilization of sedDNA (Supplementary Figure S3), which indicates that the relative level of Trex1, Trex2, and other nucleases may impact sedDNA stability in a cell type-specific manner. Furthermore, because many of these gene products analyzed in our screen function in other well-studied aspects of DNA metabolism (22), it is possible that the effects of knockdown may be indirect by inducing additional genotoxic stress that overwhelm different DNA repair pathways and DNA damage responses and/or titrate away essential NER factors such as RPA (35–37). Thus, future work will be necessary to determine the mechanisms by which these other nucleases regulate sedDNA abundance and NER.

The identification of Trex1 as a regulator of sedDNA stability is perhaps not too surprising given its known role in degrading other sources of DNA in the cell (24,25). Where TREX1-mediated sedDNA degradation takes place in the cell is not clear. Though TREX1 has been reported to primarily localize to the ER and perinuclear space (38,39), other studies have shown that it enters the nucleus in cells containing DNA damage (39,40). Using subcellular fractionation to separate soluble nuclear and cytosolic proteins from chromatin and nuclear matrix proteins, both TREX1 and the majority of the sedDNAs can be found in the apparent cytosolic fraction (Supplementary Figure S8). However, given that the sedDNA binding protein RPA readily leaks from nuclei even under mild lysis conditions (4,41,42), the localization of sedDNAs to the cytosolic fraction in this experiment is likely a result of disruption to nuclear membrane permeability. The lack of available tools for visualizing UV photoproduct-containing sedDNAs in cells in vivo over the background of UV-damaged genomic DNA has greatly limited the understanding of sedDNA processing by TREX1 and other factors, and thus future development of tools for detecting sedDNAs in vivo will be valuable. Nonetheless, it is tempting to speculate that the inefficient degradation of UV-generated sedDNAs and leakage from the nucleus may stimulate cGAS and/or other cytosolic DNA sensor pathways that TREX1 is known to suppress to limit inflammation and autoimmunity (24,29,43).

A final important observation from our work here is that Trex1 is inhibited by UV photoproducts in ssDNA (Figure 6F), and thus Trex1 only degrades the 4–7 nt located 3′ of the lesion in the sedDNA. There are also modest differences in the extent of degradation depending on the nature of the photoproduct (Figure 6G). Moreover, the presence of a UV photoproduct at the 3′ end of ssDNA significantly inhibits labeling by terminal transferase (Supplementary Figure S4) to a greater extent for (6-4)PPs than for CPDs (Figure 6I). Thus, previous studies over the past decade that have used terminal transferase to label and detect the sedDNA products of NER (3,13,17–19,30) are only observing the sedDNAs that have not yet been degraded at the 3′ end by TREX1.

Because knockdown (Figures 1–4) or knockout (Supplementary Figure S1) of Trex1 is associated with an approximate two-fold increase in sedDNA levels, our work here indicates that only roughly half of the sedDNAs that are present in the cell following exposure to UV or other UV mimetic agents are detectable with this standard labeling and detection methodology. This information should therefore be taken into consideration when quantifying NER using the detection of sedDNAs by 3′ end labeling as an assay of NER. Moreover, as genomic DNA containing UV photoproducts may leak from the nucleus and activate cytosolic DNA sensors (29,32,43), a process that is known to be suppressed by TREX1, it is possible that these other DNAs also contain UV photoproducts at their 3′ ends. Thus, our observations here may have important implications for understanding the diverse mechanisms by which UV radiation influences innate immune and other inflammatory processes that involve DNA.

## DATA AVAILABILITY

The data underlying the results are available upon request.

## Supplementary Material

gkac214_Supplemental_File
