# Supplementary material for: TREX1 degrades the 3′ end of the small DNA oligonucleotide products of nucleotide excision repair in human cells
Source: Nucleic Acids Res. 2022 Mar 31;50(7):3974–84. doi: 10.1093/nar/gkac214 (PMC9023299; doi:10.1093/nar/gkac214)
Supplement: gkac214_Supplemental_File [file gkac214_supplemental_file.pdf]

Supplementary Materials and Methods

Cell culture and vectors

Trex1-knockout (Trex1 KO) mouse embryonic fibroblasts were obtained from the Fred Perrino (Wake Forest University) and cultured in Dulbecco's modified Eagle's medium supplemented with 10% fetal bovine serum. The mammalian expression plasmids expressing Trex2 (Plasmid #40210, #79246, #44024), were obtained from Addgene.

Immunofluorescence

Cells grown on a black 24-well plate with flat and clear bottom (Ibidi) were exposed to UVC and subjected to the fluorescent labeling with anti-(6-4)PP or anti-CPD antibody (Cosmo Bio) according to the manufacturer's instructions. The fluorescence images were acquired using an automated microscope (Lionheart FX, BioTek, Winooski, VT, USA) with a 10X objective and analyzed with Gen5 software (BioTek).

Cell Survival Assays

To measure cell survival after UV irradiation, resazurin reduction assays were performed. Cells were transfected with the indicated siRNAs, incubated for 48 h, and then seeded into 96-well plates at a density of 1.5×10^4 cells. After 24 h, the cells were exposed to the indicated doses of UVC and incubated for 24 h. The number of viable cells were then determined using Resazurin Assay Kit (Abcam) according to the manufacturer's protocol. The fluorescent signals were then recorded at 550 nm excitation and 590 nm emission wavelengths using a fluorescent plate reader (Synergy HTX, BioTek). For a colony formation assay, 200-400 cells were plated in 6-well plates prior to UV irradiation and allowed to grow 7-10 days. Colonies were stained with crystal violet and counted.

Subcellular Fractionation

Following UV irradiation, cells were harvested at the indicated time points and subjected to subcellular fractionation using the NE-PER nuclear and cytoplasmic extraction kit (Thermo Fisher Scientific) as directed by the manufacturer. The fractionated extracts were then used for sedDNA isolation and detection as described in the Materials and Methods section.

Labeling of model DNA substrates

A 26-nt-long DNA oligonucleotide (AGATAACGCATGCATGCATCGCGCGC) containing a 5'-FAM label (Integrated DNA Technologies) and a single dipyrimidine sequence was exposed to 100 kJ/m^2 of 254 nm UVC radiation and then digested or not with T4 DNA Polymerase (NEB). The 26-mer and 20-mer DNAs were then excised from a 12% urea polyacrylamide gel, purified, and subjected to labeling with terminal transferase (NEB) and biotin-11-dUTP (ThermoFisher). After ethanol precipitation, the DNAs were separated on a 12% urea polyacrylamide gel, transferred to a nylon membrane, crosslinked with UVC radiation in a Stratalinker, and then probed with poly-HRP streptavidin. Chemiluminescence and fluorescence was detected on Molecular Imager Chemi-Doc XRS+ imaging system (Bio-Rad).

Supplementary Data

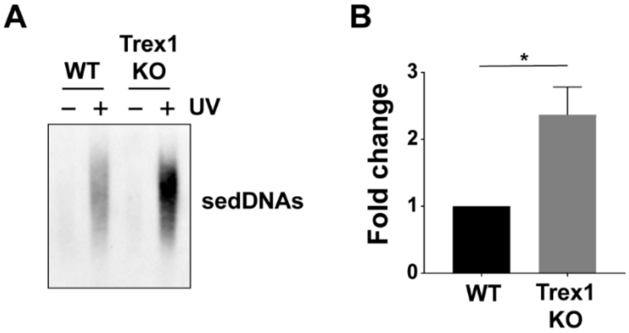

Supplementary Figure 1. sedDNA levels are elevated in UV-irradiated mouse embryonic fibroblasts from Trex1 knockout mice. (A) Spontaneously immortalized embryonic fibroblasts from wild-type (WT) or Trex1-knockout (Trex1 KO) mice were exposed to 20 J/m^2 UV radiation and then processed for sedDNAs 1 hr later. **(B)** Quantitation of sedDNAs from three independent experiments performed as in (A). The average and standard deviation are indicated (*, $p<0.05$).

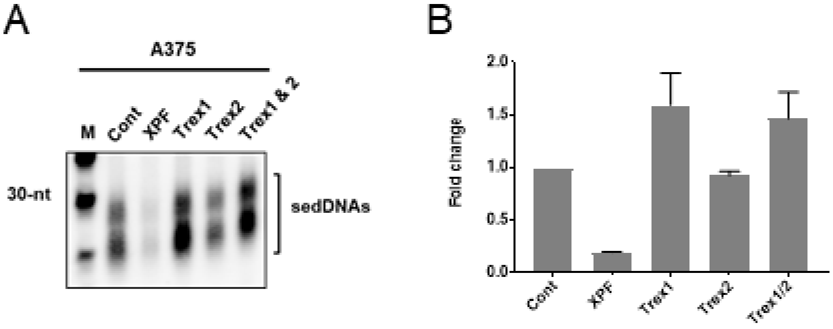

Supplementary Figure 2. Knockdown of Trex2 in A375 melanoma cells does not impact sedDNA abundance. (A) A735 cells were transfected with the indicated siRNAs, exposed to UV radiation, and then processed for detection of sedDNAs. **(B)** Quantitation of sedDNAs from two independent experiments performed as in (A).

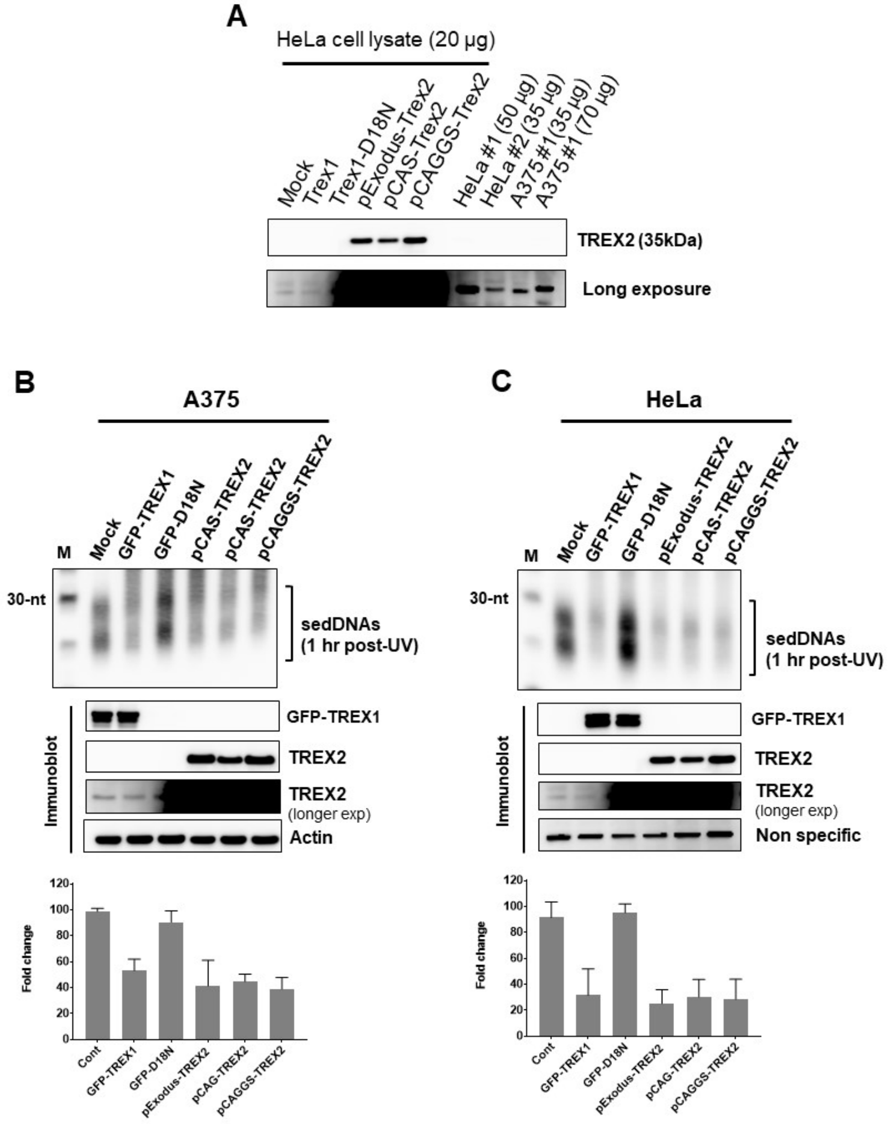

Supplementary Figure 3. Overexpression of Trex2 promotes sedDNA degradation. (A) Trex2 protein levels were detected in HeLa cells transfected with the indicated Trex2 expression vector in in A375 cells. **(B, C)** A375 and HeLa cells were transfected with vectors expressing the indicated proteins, exposed to UV radiation, and then processed for sedDNA detection. The graph shows the quantitation of sedDNAs from two independent experiments.

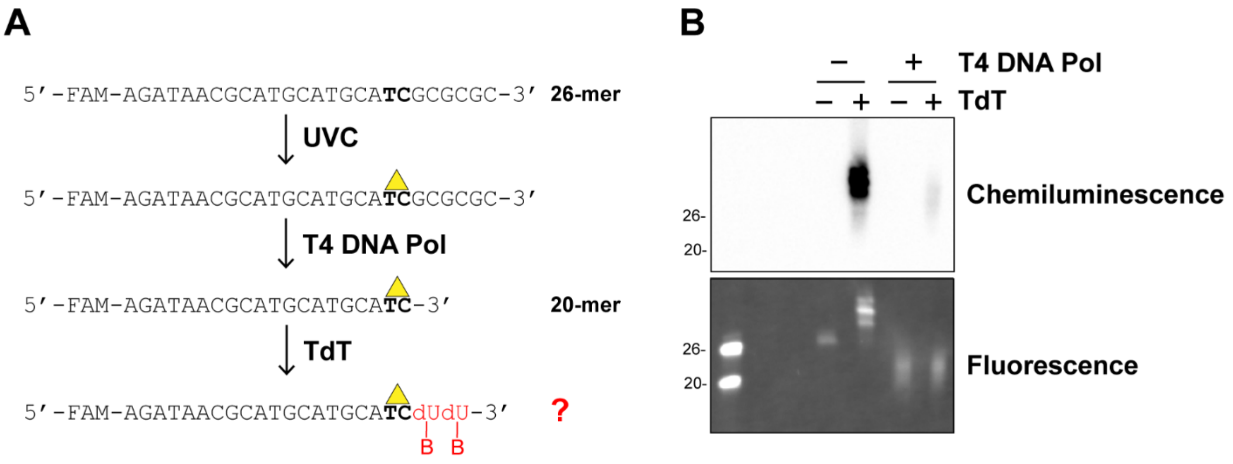

Supplementary Figure 4. Terminal transferase does not efficiently label a DNA substrate with a UV photoproduct at its 3' terminus. (A) Schematic to examine the effect of a 3' UV photoproduct on 3' end labeling by terminal transferase. The 26-mer DNA containing a 5'-FAM and single dipyrimidine (TC) sequence was exposed to UVC radiation and then digested with T4 DNA polymerase, which has exonuclease activity that is inhibited by UV photoproducts. The 20-mer product of T4 DNA Pol was gel-purified and then subjected to labeling with terminal transferase (TdT) and biotin-11-dUTP. DNAs were then examined by urea-PAGE, transferred to nylon, and then detected with both fluorescence and chemluminescence with strepatividin-HRP. **(B)** Whereas TdT can readily 3 'end label the UV-irradiated DNA substrate that is not digested with T4 DNA Pol, it is unable to efficiently do so on the T4 DNA Pol-digested substrate.

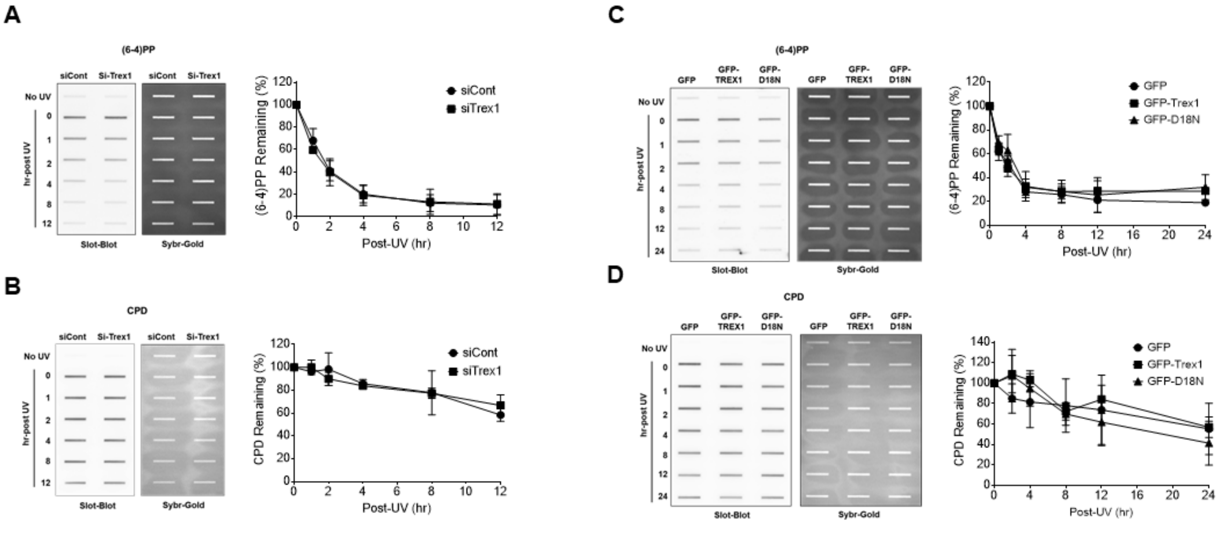

Supplementary Figure 5. Knockdown and overexpression of Trex1 does not impact UV photoproduct removal from genomic DNA. (A) Genomic DNA was purified from UV-irradiated HeLa cells transfected with control or Trex1 siRNA at the indicated time points. DNA was immunoblotted and probed with anti-(6-4)PP antibody. The graph shows the average level of unrepaired (6-4)PPs at each time point from 3 independent

experiments. **(B)** Cells were analyzed as in (A) but for CPD content in genomic DNA. **(C)** Genomic DNA was purified from UV-irradiated HeLa cells transfected with the indicated GFP fusion proteins and probed with anti-(6-4)PP antibody. The graph shows the average level of unrepaired (6-4)PPs at each time point from more than 3 independent experiments. **(D)** Cells were analyzed as in (C) except for CPD content in genomic DNA.

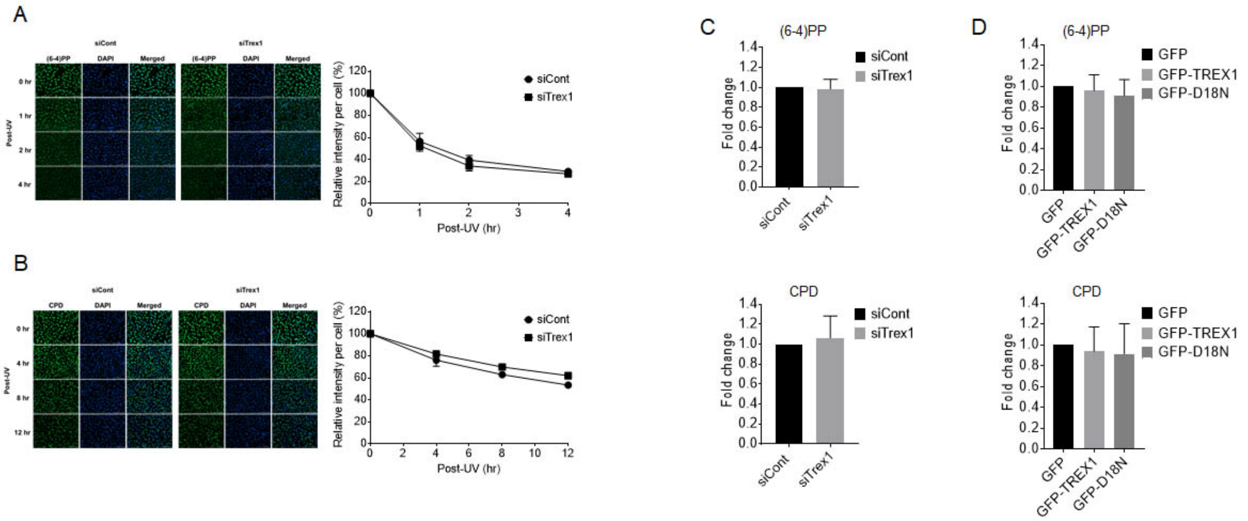

Supplementary Figure 6. Knockdown and overexpression of Trex1 does not impact UV photoproduct removal from genomic DNA. (A, B) Immunofluorescence microscopy was used to detect (6-4)PP and CPD content in cells treated as in Supplementary Figure 5 but processed for microscopy. The graphs show the average fluorescence intensity normalized to cells fixed and stained immediately after UV exposure. **(C, D)** Quantitation of the immunoslot blot data at 0 hr time points following UV irradiation in Supplementary Figure 5.

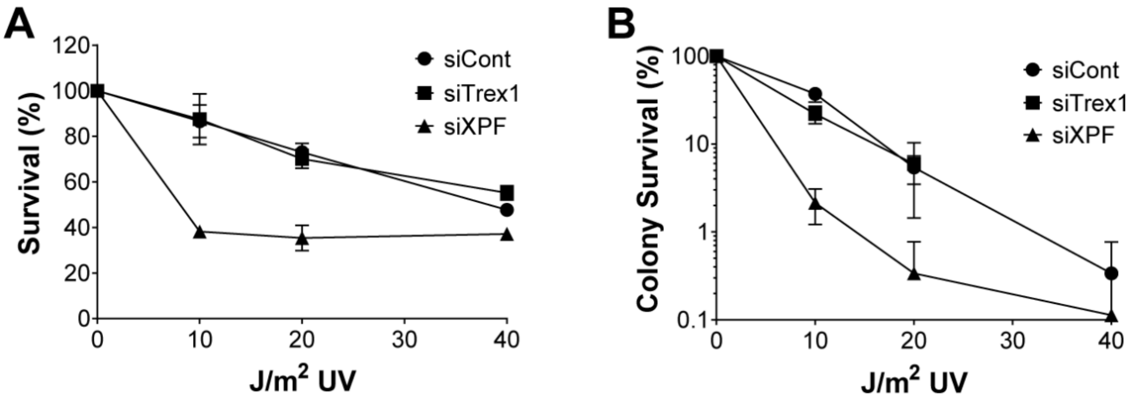

Supplementary Figure 7. Knockdown of Trex1 has no effect on the survival of UV-irradiated cells. (A) A375 cells were transfected siRNAs targeting the indicated genes,

exposed to UV, and then subjected to a resazurin-based cell viability assay. **(B)** Cells were treated as in (A) except cell survival was measured with a clonogenic cell survival assay.

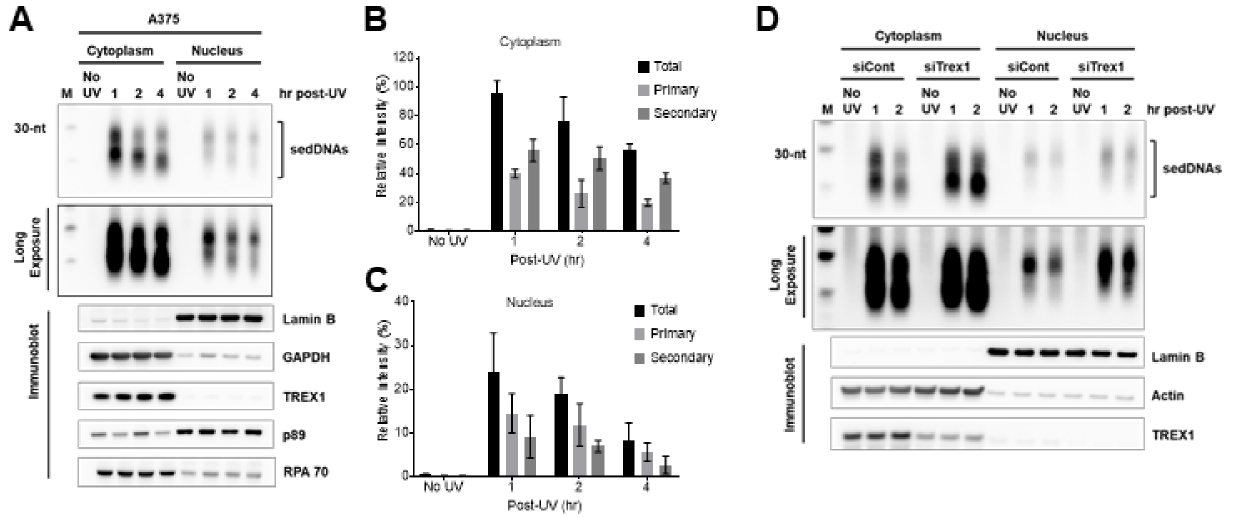

Supplementary Figure 8. Localization of sedDNAs as determined by subcellular fractionation. (A) A375 cells were exposed to UV, and then subjected to subcellular fractionation, and then processed for analysis of sedDNAs. Fractionated cell lysates were immunoblotted for the indicated proteins. **(B, C)** The graphs show the average level of total, primary, and secondary sedDNAs from the cytoplasmic and nuclear sedDNAs. **(D)** Knockdown of Trex1 results in increased sedDNAs in both the cytoplasmic and nuclear fractions.
